# Sustained sustainable development actions of China from 1986 to 2020

**DOI:** 10.1038/s41598-021-87376-8

**Published:** 2021-04-13

**Authors:** Bingsheng Liu, Tao Wang, Jiaming Zhang, Xiaoming Wang, Yuan Chang, Dongping Fang, Mengjun Yang, Xinzhang Sun

**Affiliations:** 1 School of Public Affairs, Chongqing University, Shapingba District, Chongqing, 400044 People’s Republic of China; 2 The State Key Lab of Cryospheric Science, Northwest Institute of Eco-Environment and Resources, Chinese Academy of Science, Lanzhou, 730013 Gansu People’s Republic of China; 3 School of Management Science and Engineering, Central University of Finance and Economics, Changping District, Beijing, 102206 People’s Republic of China; 4 School of Civil Engineering, Tsinghua University, Haidian District, Beijing, 100084 People’s Republic of China; 5 The Administrative Center for China’s Agenda 21, Haidian District, Beijing, 100038 People’s Republic of China

**Keywords:** Environmental social sciences, Sustainability

## Abstract

Achieving the Sustainable Development Goals (SDGs) is a long-term task, which puts forward high requirements on the sustainability of related policies and actions. Using the text analysis method, we analyze the China National Sustainable Communities (CNSCs) policy implemented over 30 years and its effects on achieving SDGs. We find that the national government needs to understand the scope of sustainable development more comprehensively, the sustained actions can produce positive effects under the right goals. The SDGs selection of local governments is affected by local development levels and resource conditions, regions with better economic foundations tend to focus on SDGs on human well-being, regions with weaker foundations show priority to basic SDGs on the economic development, infrastructures and industrialization.

## Introduction

In 2015, 193 countries of the United Nations General Assembly adopted the 2030 Agenda for Sustainable Development in New York^1^, which has become the program for most countries around the world to implement sustainable development. However, the SDG Summit in 2019 pointed out that the world was not on track to achieving the SDGs by 2030, and global progress in some areas of sustainable development had either stagnated or been reversed^2^. The COVID-19 outbreak in 2020 has evolved into global public health and economic crisis, which has had severe negative impacts on most SDGs. And poor countries and vulnerable groups will be hit hardest in the long run^3^. Although many countries are developing and implementing policies to support sustainable development, new policies often bear the significant mark of the current government. It is a common phenomenon that national policies and strategies change with changes in government or leadership; for example, different governments in the United States and Australia have distinct attitudes towards climate change response policies aimed at SDG13 ^4^. Policy instability or “mobilized governance” will lead to a negative effect on sustainable development^5–8^, and maintaining the “sustainability” of sustainable development policy itself is the premise for achieving SDGs.

Existing studies about SDGs can be mainly categorized into two groups:

One group of research is debating on the scientific rationality of SDGs. Some researchers believe that the SDGs reflect the ideology of Anthropocentrism, which is intertwined with the practice of industrialization and the ideology of economic growth^9,10^. Sam Adelman argued that the SDGs promoted a weak, anthropocentric form of sustainable development that ignored ecological reality and continued to prioritize economic growth above social justice and environmental protection^11^. Undeniably, the majority of sustainable development models still suffer from ‘an insufficiently developed theoretical framework’^12^, but with the deepening of the theoretical research, the concept of environmental limit, carrying capacity, and planetary boundaries have been gradually fulfilled. For instance, some studies maintain that the SDGs should be integrated with the planetary boundaries so as to pursue the prosperity of human society within the limits of natural capital^13^. Holden et al. proposed a three-imperatives model for sustainable development including satisfying human needs, ensuring social equity, and respecting environmental limits, and highlighted the equally essential role of the three imperatives^14^. Comparatively, economic growth was just a potential means to fulfill primary dimensions, rather than a primary dimension of sustainable development^14,15^.

The other research cluster primarily focuses on assessing the trade-offs and synergies between SDGs^16–18^. For example, researchers have identified the prominent trade-offs between SDG12 and other SDGs, while extensive synergies between SDG3 and other goals have also been recognized^18^. Despite the overlaps and conflicts among SDGs, researchers had proved that the holistic way of thinking and the network attribute of SDGs would secure better outcomes for each goal. The SDGs network may not be completely self-consistent from a mathematical perspective, but targeted direct efforts and policies are able to maximize the benefit of SDGs under complex reality constraint^19^. Crist et al. also maintained that, although the social and economic development level vary enormously across countries, policy and regulation are priority tools for each country to leverage so as to guide population change toward an ecologically and socially sustainable direction^20^. Therefore, it is quite essential to assess the effect of sustained policies on achieving SDGs.

Much have done about assessing the progress and current conditions of all 17 SDGs^21–23^, however, research on stable sustainable development policy for an extended period and its implementation is lacking, and the characteristics of sustainable development actions over a longtime span remain to be disclosed. The policy of the China National Sustainable Communities (CNSCs) led by the Ministry of Science and Technology (MOST) has been implemented since 1986 and has become the experiments and demonstrations of innovation-driven sustainable development^24,25^. The policy has been implemented by four generations of state leaders for more than 30 years, which can be regarded as sustained sustainable development actions. Through January 2020, there have been 189 sustainable communities distributed widely in eastern, central and western China, covering cities with different natural environments, resource endowments and economic levels. Therefore, studying the effect of the CNSCs policy on the achievement of SDGs will be of reference significance for the implementation of sustainable development in different countries around the world.

The effect of long-term policies could be evaluated by several quantitative modeling approaches, such as general equilibrium model^26^, difference-in-difference model^27^, and input–output model^28,29^. However, these methods have high data requirements, which are usually difficult to satisfy by existing statistical system of a nation or city. The text analysis (TA) is an alternative approach to extracting the insight of texts (i.e., policy documents, reports, and the literature in a particular filed) and their objectives^30^, and has been widely used in policy analyses: (1) TA is used to define a certain concept or clarify the concept’s origin, rationale, and development state^31^; (2) the method is employed to identify specific actions in policy implementation, so as to find the critical factors or practices to policy success^32–36^; and (3) TA is applicable to evaluating policy performance and status quo^37,38^. Compared with other quantitative methods, TA has the following advantages: first, the text materials related to policy formulation and implementation are easier to obtain; second, the analysis process is simple and reversible; third, the dynamic process and details of policy implementation can be reflected, enabling policy makers to summarize useful experience.

This study applies the TA method to illustrate the construction process and achievements of CNSCs from the spatiotemporal dimension, focusing on questions about three aspects: (a) the SDGs that the sustainable communities prefer; (b) the spatiotemporal changing trend of these sustainable communities since 1986 and the sustainability of the policy; (c) the performance of sustainable communities and the effect of the CNSCs policy on achieving SDGs. To answer these questions, we applied data from the evaluation for 189 sustainable communities in 2018 by the MOST. The construction themes and attainments were extracted, sorted and mapped to the 17 SDGs and 169 targets. Then, the spatiotemporal analysis was carried out to find the relationship between the action themes and factors in politics, regions, economy, society and environment and draw some conclusions.

## Results

### The distribution of sustainable communities

**Figure 1:**
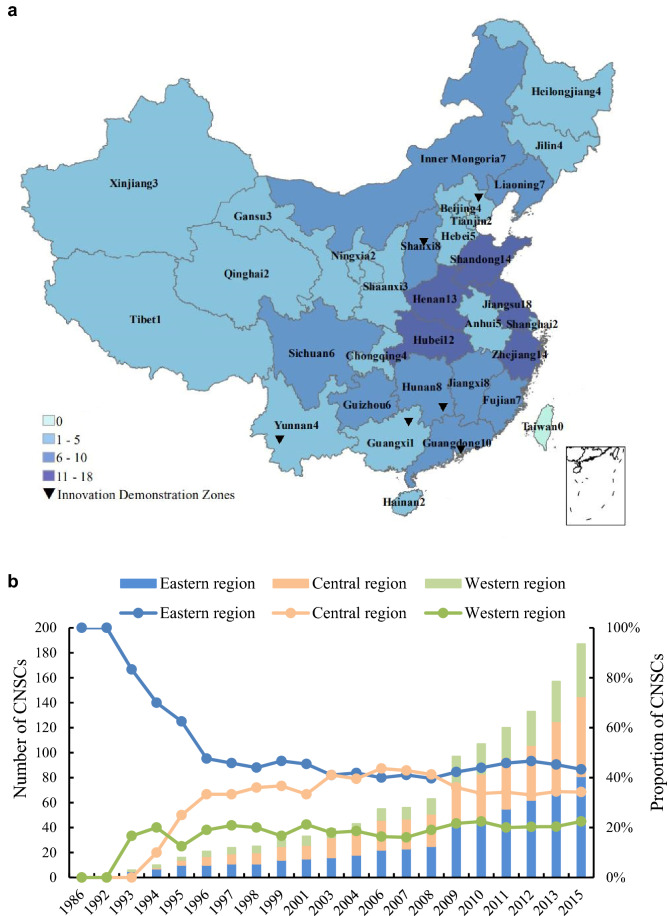
The distribution and change of CNSCs and IDZSDs. (**a**) The distribution of CNSCs and IDZSDs. The data for the base map were derived from the Resource and Environment Data Cloud Platform^39^, the map was generated by ArcGIS 10.4.1 (https://desktop.arcgis.com/zh-cn/arcmap/10.4/get-started/installation-guide/introduction.htm), and the number in the figure legend denotes the number of CNSCs in each province. (**b**) Change in the number of CNSCs.

China constructed a total of 21 batches of 189 CNSCs from 1986 to 2015, covering 31 provinces (Fig. 1a). The cities, districts, counties and townships accounted for 15%, 34%, 48% and 3% of the CNSCs, respectively; the number of CNSCs in eastern coastal areas was the greatest, and it was followed by the number in the central provinces, with the lowest number in the western provinces. The number of CNSCs increased significantly after 2008 (Fig. 1b), showing that the Chinese government has fully enforced the construction of CNSCs for more than 30 years and attached more importance since 2008. The government initially established these sustainable communities in the eastern region, where the economic development pace is faster, and then they expanded into the central and western regions; now, the ratio of the number in eastern, central and western regions is 5:3:2, respectively, which reflects that China has gradually strengthened the regional equality in the process of policy implementation. In 2015, the UN convened the Development Summit and adopted the 2030 Agenda. In response, China suspended the construction of CNSCs and switched to the Innovation Demonstration Zones for Implementing the 2030 Agenda of Sustainable Development (IDZSDs), which were based on prefecture-level cities. After two years of planning and preparation, China identified 2 batches of 6 innovation demonstration zones in 2018 and 2019, uniformly distributed in the eastern, central and western regions, and more than half of the IDZSDs are small cities with relatively low economic level but good development experience.

### The selection preference of SDGs in sustainable communities

**Figure 2:**
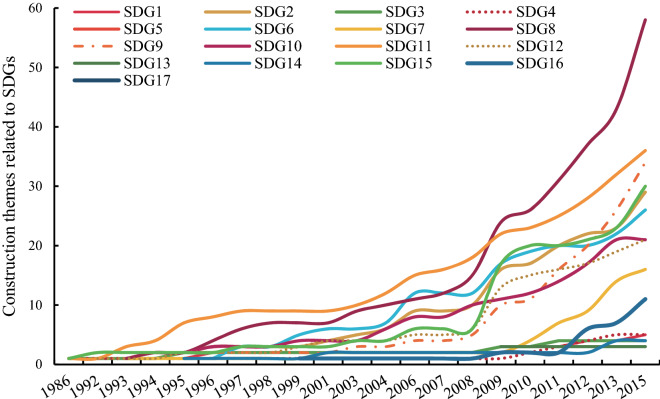
The number of construction themes related to different SDGs in CNSCs.

We analyzed the major themes for the sustainable communities and found that these construction themes cover 15 of the 17 SDGs (Fig. 2), excluding SDG5 (Gender equality) and SDG17 (Partnerships for the goals). The possible reason is that “gender equality” was written into the Constitution of the People’s Republic of China as early as 1954, and China has made some progress in promoting equal access to education, employment opportunities and political participation between men and women. Therefore, gender equality is no longer a major social issue that Chinese local governments focus on. Since the reform and opening up in 1978, China has constantly enhanced external communication in economy and culture. Numerous sustainable communities established sister city relationships with foreign cities, but the partnership was often regarded as a means to achieve goals rather than construction themes. Among the 15 SDGs covered by construction themes, most were related to SDG 8 (Decent work and economic growth) and SDG 11 (Sustainable cities and communities), showing that China has attached great importance to economic development and urban infrastructure construction in the past 30 years. The themes related to SDG 9 (Industry, innovation and infrastructure) increased rapidly after 2008, which can be attributed to the industrial structural adjustment in response to financial crises and the impact caused by natural disasters such as the Wenchuan earthquake and snow calamity. The numbers of SDG 6 (Clean water and sanitation), SDG 2 (Zero hunger), SDG 15 (Life on land), SDG 12 (Responsible consumption and production), SDG10 (Reduced inequalities) and SDG 7 (Affordable and clean energy) were at a medium level, and the numbers of SDG 16 (Peace, justice and strong institutions), SDG 4 (Quality education), SDG 14 (Life below water), SDG 1 (No poverty), SDG 3 (Good health and well-being) and SDG 13 (Climate action) were at a lower level. Waage divided all 17 SDGs into three concentric layers, including well-being, infrastructure and the natural environment^40^. According to this classification, the sustainable communities preferred the themes about the infrastructure layer, which aims to provide products and services for achieving well-being (SDG 2, SDG 6, SDG 7, SDG 8, SDG 9, SDG11 and SDG 12), while the themes related to well-being itself (SDG 1, SDG 3, SDG 4, SDG 5, SDG 10 and SDG 16) were rarer. The deviant results reflected the prevalent understanding variance of the Chinese city governments regarding the conceptual scope of sustainable development. The governments believed that the meaning of sustainable development lied in the support of infrastructure rather than the direct governance of well-being-related issues. In fact, China has made great efforts in poverty alleviation, education and medical care. For example, China has worked on poverty reduction for a long time, formulating the “precision poverty alleviation” policy, with the goal of eliminating absolute poverty by 2020 through a series of actions. Nine-year compulsory education has been in place in China for more than 40 years; as of 2017, the net enrollment rate of school-age children and the gross enrollment of secondary school reached 99.9% and 100%, respectively^41^. The medical system of China has improved, and insurance coverage has stabilized at more than 95%. Moreover, the effective response to the COVID-19 pandemic in early 2020 also reflected the medical proficiency level of China. In the natural environment-related themes (SDG 13, SDG 14 and SDG 15), the number of SDG 15 was larger than that of SDG 13 and SDG 14, which might result from the fact that most cities are inland cities. At the same time, these numbers showed that local cities paid less attention to the concept of climate change before 2015. Actually, China has made great efforts to tackle climate change since it signed the Paris Agreement.

### The changing trend of sustainable communities

**Figure 3:**
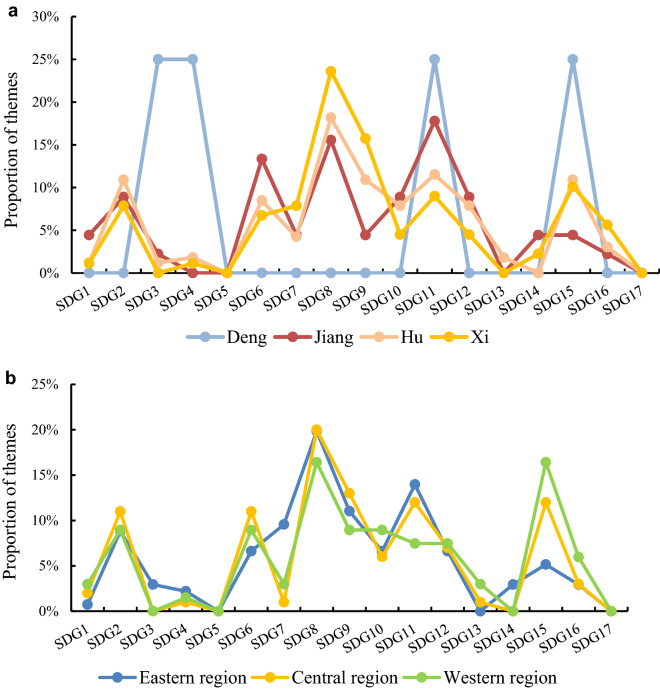
The proportion of themes in different periods or regions. (**a**) The proportion of themes in different periods. (**b**) The proportion of themes in different regions.

In temporal distribution (Fig. 3a), China has experienced four generations of state leaders since the first sustainable community was established in 1986. There were only 2 sustainable communities making attempts in health, education, city infrastructures and land environment protection under the leadership of Deng (1978–1989). During the leadership of Jiang, Hu and Xi, the number of themes related to different SDGs maintained a similar proportion. The proportion of themes in the aspect of economic growth and industrial innovation (SDG 8 and SDG 9) increased gradually, which might also indicate the reason for the constant economic level rise over 30 years. Although the proportion of infrastructure construction (SDG 11) remained at a relatively high level, it continued to decline with the change in state leaders, indicating that the status of city infrastructure construction as a local development characteristic was declining. The proportion of themes on SDG 15 and SDG 16 had an upward trend, which means that the governments strengthened the conception of inland environmental protection and law-based governance.

In the regional distribution (Fig. 3b), themes on SDG 8, SDG 9 and SDG 11 had higher selection proportions in the eastern, central and western regions. In comparison, the inland central and western regions with relatively weak economic bases and ecosystems showed obvious motivation for achieving SDG 1, SDG 13 and SDG 15, while the eastern coastal region with a better economic foundation explored SDG 3, SDG 7 and SDG 14 more. Therefore, it can be seen that the conditions regarding resources, the environment and the economy had a certain effect on the priority of achieving SDGs.

### The performance of sustainable communities

**Figure 4:**
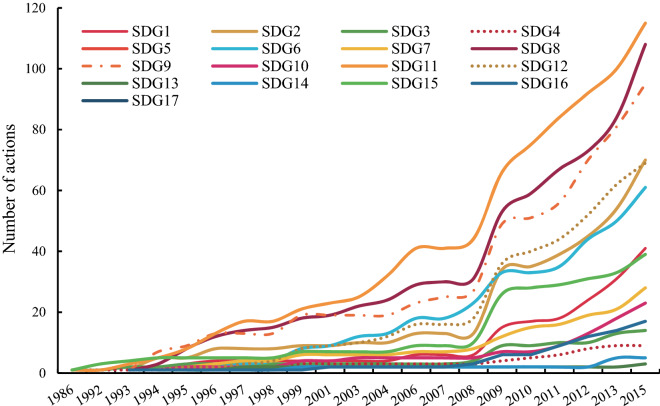
The number of actions related to different SDGs in CNSCs.

After the sustainable communities were set up, local governments incorporated the construction themes into their development plans and programs, carrying out related work. According to a 2018 evaluation report by MOST on the effect of 189 sustainable communities, 168 (89%) sustainable communities had fully implemented the construction work and had obtained achievements, 11 (6%) sustainable communities had failed to achieve the goals of development plans, and 10 sustainable communities (5%) were not included in the evaluation due to administrative division adjustments and other reasons, which can be regarded as invalid data. The results of the evaluation report illustrated that, as a policy mechanism for exploring the way of sustainable development, the policy of CNSCs had both successful and failed cases, and the proportion of successful cases accounted for the vast majority, which showed strong enforceability. The number of actions to achieve goals was generally the same as the number of themes (Fig. 4); actions on SDG 11, SDG 8 and SDG 9 were the most common, which indicated a higher consistency between the plans and actions of the CNSCs policy. Meanwhile, the number of actions related to SDGs was larger than the themes approved at the beginning, probably because of the improvement in the understanding of the sustainable development concept scope and the willingness to implement SDGs in more extensive contexts. It was noteworthy that the actions of some SDGs had a higher proportion than themes on those SDGs, such as for SDG 1. The actions toward poverty alleviation were concentrated in the sustainable communities established after 2012, corresponding to the “precision poverty alleviation” proposed in 2013.

### The effect of CNSCs policy on achieving SDGs

A particular bias is that the achievement of SDGs is rooted in scientific theories, rather than political resonances. In other words, assessing the technological feasibility of SDGs is more strongly representative than the evidence of political will. Dawes discussed the effect of “science” and “policy” on achieving SDGs respectively: in SDGs network, technological solutions in an area linked to one goal can drive improvements of other goals in the system, and the “science” is itself aligned with the intrinsic and systemic effects within the network; while the “policy” accords more obviously with extrinsic effects, and the direct investment of governments or civil society can solve local issues related to specific goals^19^. Thus, the conflicts between SDGs and their associated negative impacts can be addressed by combining technology and targeted policies.

This paper focused on gauging the role of a sustained policy in achieving SDGs in China, we rated the number of actions of sustainable communities towards SDGs and compared them with the dashboard for SDG implementation in China, which was released by Sustainable Development Solution Networks (see Table 1). The results proved that, as a whole, the SDGs to which sustainable communities paid more attention and into which they put more effort showed a better current situation; conversely, other SDGs gained less attention, and implementation actions were performed more poorly. The differences between the number of actions and the current situation were within one grade. The reason for the differences was that the policy was led and implemented by the MOST of China, and the themes and implementation actions tended to be more science- and technology-oriented. To some extent, the number of actions can represent the input of sustainable communities to each SDG, while it may produce deviations because of less attention to the goals on human well-being. For example, education (SDG 4) was the SDG with the highest disparity in grades, as previously analyzed, possibly because the nine-year compulsory education requirement had been carried out for more than 40 years, and local governments did not realize that education also belonged to the scope of sustainable development. Additionally, attention and actions towards infrastructure construction (SDG 11) and industrial innovation (SDG 9) were at a high level, while China still faced significant challenges in achieving the two SDGs. This may result from the obvious distinctions in the infrastructure and industrialization level between the eastern and western regions, and constant input into these SDGs was still required.

**Table 1:** Comparison of the number of actions in sustainable communities and the progress of achieving SDGs in China.

SDG	1	2	3	4	5	6	7	8	9	10	11	12	13	14	15	16	17
SDG dashboard for China	A	C	C	A	C	C	C	A	C	D	C	B	B	D	C	C	B
Grade of number of actions on SDGs	B	B	D	D	D	B	C	A	A	C	A	B	D	D	C	D	D

## Discussion

Sustainable development has gone through Agenda 21 (1992), Millennium Development Goals (2000) and sustainable development goals (2015), while China has conducted experiments and practices at the governmental level since 1986. We aimed to provide China’s experience in achieving SDGs to other countries.

The policy of CNSCs has been implemented for almost 30 years, and it can be regarded as a long-term sustainable development practice of a country after the introduction of market mechanism. Therefore, the policy and its results are representative. To evaluate the effect of the CNSCs policy, this paper employed a qualitative TA method to extract information and classify themes from MOST’s reports on 174 CNSCs, and conducted statistical analyses for CNSCs’ SDGs and relevant actions. The text analytics framework developed by this paper provides an open and reversible approach to policy analysis, which substantially reduces the need for quantitative data and allows more in-depth analyses as sustainable development theory continues to evolve and more data of CNSCs become availability in the future.

More local governments have been willing to choose economic development (SDG 8) as one of the themes, which matches the national situation of China as a developing country. For countries with different levels of socio-economic development and hence having different opportunities and constraints, their sustainable development priorities vary^12^. As discussed in Holden et al. (2017), some countries may satisfy the imperatives of respecting environmental limits and ensuring social equity, but fail to meet human basic need. This makes extreme poverty eradication and human capacities enhancement priorities of their policy management. As a result, policies and institutions that promote economic growth are essential for these countries’ capacity building^14^. The preference of theme selection for different regions implied that areas with solid economic foundations focused more on the SDGs requiring high technologies and massive investment, such as health, well-being and sustainable energy. To some extent, it suggested that a better economic foundation can provide stronger support for cities to solve social and environmental problems, and economic growth is not the opposite of sustainable development. Under the leadership of the Chinese government with Xi at its core, more sustainable communities chose inclusive economic growth, infrastructure, and innovation (SDG 8 and SDG 9) as construction themes, and these themes have regional or even global spillover effects. Corresponding national development strategies, such as the “Belt and Road Initiative” and “Made in China 2025”, will help China to promote the achievement of SDGs on regional and global scales, generalizing its excellent experience and embodying the development concept of a community with a shared future.

China prioritized economic growth in the past, and its extensive economic growth once posed environmental pressure, especially in the eastern regions. But the Chinese government has learned the lessons, and variations in the CNSCs’ themes have also reflected that the government has recognized sustainable development in a more holistic and systematic manner. In the period of Hu and Xi, although the SDGs related to basic conditions were still the largest part in the construction themes, the number of themes related to human well-being and the environment was also increasing. Enriching the connotation and scope of sustainable development requires the government to incorporate them into the national basic policy and to influence the selection of local levels from top to bottom. Since the reform and opening up (1978), the national development concept has gone through five stages: the “focus on economic construction” (1980), “emphasis on both material and spiritual civilizations” (1982), “trinity of political, economic and social construction” (1986), “construction of politics, economy, culture and society" (2005) and “five in one of economic, political, cultural, social and ecological civilization construction” (2012). These social and ecological civilization constructions in China manifest the central government’s implementation of the sustainable development concept. Policies such as precision poverty alleviation and ecological redlines and the conviction that “lucid waters and lush mountains are invaluable assets” reflect the country’s determination to achieve SDGs and to build a 'Beautiful China'. Economic growth underpins a prosperous society, and is thus a major cornerstone that forms the foundation of sustainable development. However, economic growth must be pursued within environmental carrying capacity. On the other hand, China has been exploring new development models in western areas with weak economic foundations and vulnerable ecological environment, such as making full use of desert areas to deploy renewable energy technologies (i.e., solar and wind farms) and build big data centers (that will be powered by renewable electricity), to achieve well-being improvement and ecological restoration simultaneously. The main purpose of the CNSCs is to make attempts and accumulate experience, and the results have demonstrated that sustained sustainable development actions can be effective under the premise of suitable goals. Generally, the more input that is given to some SDGs, the better the country will perform in those SDGs. Local governments with lower economic levels tend to prioritize the economy, infrastructure and industrialization. With the growth in the economy, the higher requirements of well-being and living conditions will force local governments to provide equal education, healthcare and political services, along with a better environment and more efficient resource exploitation. Moreover, comprehensively understanding the concept of sustainable development and incorporating it into national policies is of great importance for the country to fully achieve SDGs. The changing of theme preference has reflected the differences existing in the understanding of sustainable development of the Chinese government in different leadership periods. Due to the imperfect understanding of the scope of sustainable development in the past, the themes and actions related SDGs on human well-being, climate and environment were fewer than those related to economic development. It is worth noting that the sustainable development concept of the national government has been constantly improved with the implementation of the CNSCs policy, and the support measures for SDGs which were less concerned in the past are also increasing, the effectiveness of these support actions remains to be discussed due to the short implementation time. These conclusions can provide references for countries around the world to implement the 2030 Agenda for Sustainable Development and achieve SDGs.

## Methods

We used a TA method to evaluate the themes and actions of sustainable communities and innovation demonstration zones. There were two main steps.

### Step 1 The processing methods of mapping the construction themes and implementation actions to the SDGs

This research mainly adopted the TA method, which makes subjective interpretation of text content through a systematic classification process of coding and identifying themes or patterns. The method is widely used in social science research and includes three means^42^: (1) conventional content analysis, where the coding categories are directly derived from text information; (2) the directed approach, which uses existing research as the basis for classification; and (3) summative content analysis, conducting frequency statistics and comparison or in-depth analysis of keywords based on context. In this study, we applied the latter two means to conduct TA on the materials of sustainable communities. The specific process was carried out as follows:

### Theme classification

We deeply understood the implementation measures corresponding to all 17 SDGs and 169 targets through “China’s National Plan on Implementation of the 2030 Agenda for Sustainable Development” and built a corpus of thematic keywords of the construction of sustainable communities. Then, we classified the themes in batches according to the keywords (see Table 2).

**Table 2:** SDG corpus corresponding to keywords.

	SDG 1	SDG 2	SDG 3	SDG 4	SDG 5	SDG 6
Keywords	Poverty alleviation; poverty relief; subsistence allowances	Agriculture; grain	Medical; health	Education	Men and women; gender	Water; sanitation
	SDG 7	SDG 8	SDG 9	SDG 10	SDG 11	SDG 12
Keywords	Low carbon; energy; energy saving	Economic transformation; transformation and upgrading; tourism	Industry	Plan as a whole integrated development	Urbanization	Circular
	SDG 13	SDG 14	SDG 15	SDG 16	SDG 17	
Keywords	Climate	Sea	Forest	Harmony; stable	International cooperation; partnership	

### Information extraction

We extracted the text verbatim and categorized the text on the implementation measures from the reports submitted by sustainable communities. The text and documents are large in volume and highly unstructured, placing high requirements on the subjective initiative, and it was not suitable to use keyword frequency statistics directly. Therefore, researchers read the evaluation opinions of the sustainable communities carefully and mapped the text information to 169 targets of SDGs in the “China’s National Plan on Implementation of the 2030 Agenda for Sustainable Development”. One action corresponded to one SDG, and the repetitive goals were counted only once.

### Discussion on classification differences

To fully overcome the subjectivity of text classification, two researchers analyzed text separately at first, and then we compared the results from the two researchers and discussed the differences with the third researcher. Finally, the classification results of themes and actions were reviewed by three experts who had experience in research related to SDGs to add missing categories, identify fuzzy theme categories and unify opinions.

In the TA, we removed 9 sustainable communities with vague themes and summarized 303 themes of 180 sustainable communities. We also removed 7 sustainable communities without submitting reports and 9 sustainable communities without specific progress, summarizing 679 actions in 174 sustainable communities. Sustainable communities would generally propose several themes and multiple actions, so the themes and actions of each sustainable community can be matched to more than one SDG.

### Step 2 Rating the number of actions on SDGs in sustainable communities and the progress of implementing SDGs in China

The rules of rating the number of actions on SDGs in the sustainable communities are shown in Table 3. The method for grading the progress of achieving SDGs in China was based on the results of the SDGs dashboard and index report issued by the United Nations Sustainable Development Solution Networks (see Table 4).

**Table 3:** Grade of number of actions on SDGs.

Number of actions on certain SDG	80–120	40–79	20–39	0–19
Grade	A	B	C	D

**Table 4:** Grade of progress in achieving SDGs.

Evaluation results of progress of achieving SDGs	SDG achieved	Challenges remain	Significant challenges	Major challenges
Grade	A	B	C	D

## Data Availability

Data generated or analyzed during the study are available from the corresponding author by request.
